# Assessing the impact of climate and control interventions on spatio-temporal malaria dynamics using a stochastic metapopulation model

**DOI:** 10.1371/journal.pcbi.1014004

**Published:** 2026-03-17

**Authors:** Alexandros Angelakis, Anton Beloconi, Bryan O. Nyawanda, Sammy Khagayi, Simon Kariuki, Stephen Munga, Patrick K. Munywoki, Godfrey Bigogo, Penelope Vounatsou

**Affiliations:** 1 Swiss Tropical and Public Health Institute, Allschwil, Switzerland; 2 University of Basel, Basel, Switzerland; 3 Center for Global Health Research, Kenya Medical Research Institute, Kisumu, Kenya; 4 United States of America Centers for Disease Control and Prevention, Nairobi, Kenya; UniversitatsSpital Zurich, SWITZERLAND

## Abstract

Despite intensive malaria control efforts, the lowlands of western Kenya continue to experience high malaria transmission. Spatial and temporal variations in climatic factors, interventions, parasite dispersal, and human travel, influence malaria incidence in moderate-to-high transmission areas. Additionally, population movement facilitates the importation of parasites from endemic to non-endemic areas, sustaining infections where local transmission would otherwise be unsustainable. The aim of this work was to develop a process-based stochastic metapopulation transmission model that accounts for key mechanisms of malaria dynamics, such as immunity, infectivity, and migration, while considering both the host and vector mobility. The model also incorporates and quantifies the effects of malaria interventions and climate variability at the local scale. Unlike existing models that often consider these drivers in isolation, our framework captures their joint influence within a single, mechanistic system. We show that, between 2008 and 2019, the developed metapopulation model accurately captured the effects of small-scale heterogeneity at the subpopulation level in western Kenya. Although demonstrated in a Kenyan context, the model is generalisable to other endemic regions and can support localized forecasting and intervention planning under future climate scenarios. Finaly, we assess its potential to forecast malaria incidence at the spatial-unit level, by integrating future climatic conditions with intervention scenarios.

## Author summary

Malaria remains a major public health challenge in the lowlands of western Kenya, where intense disease transmission persists despite ongoing control efforts. This persistence is driven by a complex inerplay of factors, including local climate and geography, population movements that spread the parasite between communities, and the varied impact of interventions like bed nets and indoor spraying. In this study, we developed a mathematical model to capture these diverse influences on malaria transmission. The model not only tracks parasite spread between humans and mosquitoes, but also accounts for communities interactions through travel and for the effects of local climate on mosquito development and disease risk. In addition, it incorporates key features of malaria dynamics, including human immunity, parasite transmissibility, and the impacts of control measures. We applied the model to data collected from 2008 to 2019 in western Kenya and found that it successfully reproduced complex, village-level variations in malaria cases. By taking into account climate trends and intervention coverage, the model can also forecast future malaria incidence under different scenarios.These findings underscore the importance of considering fine-scale heterogeneity and population movement when designing more effective, targeted strategies to reduce the malaria burden.

## Introduction

Malaria continues to pose a global public health challenge, despite increased implementation of intervention strategies. In 2023, an estimated 263 million malaria cases and 597000 deaths were reported worldwide, with approximately 94% of cases and 95% of deaths occuring in the African Region [1]. Most malaria-related deaths (around 78%) occurred among children under the age of five. Recent models suggest a possible expansion in the global population exposed to the risk of malaria [2,3], while others propose a shift in the spatial distribution of global malaria incidence [4]. This highlights the necessity of accurate spatio-temporal malaria forecasting models. Around 70% of the population in Kenya is at risk of malaria [5]. The implementation of insecticide treated nets and indoor residual spraying, case management and the pilot introduction of malaria vaccines in 2019 reduced parasite prevalence from 8% in 2015 to 5.6% in 2020 [7,6]. However, the rate of reduction has slowed since 2017, and the disruption of malaria services caused by COVID-19 highlights the importance of implementing alternative and long-term preventive measures to effectively reduce malaria prevalence in Kenya.

The impact of climatic factors on mosquito life cycle, and malaria transmission have been extensively documented. Climatic factors influence various components, including mosquito larvae development time, mosquito survival rate, and malaria parasite development in infected mosquitoes [8]. An increase in temperature up to 30°C can enhance vector metabolic rate, egg production, and the frequency of blood meals [9]. However, temperatures below 16 or above 34°C may be detrimental to mosquito populations and parasite development [10]. Temperature has a non-linear effect on malaria incidence, with ideal temperatures ranging from 20 to 27°C for an adult mosquito and the parasite [8,11–14]. Rainfall directly impacts vector abundance by creating favorable wet conditions for larval development and adult abundance, which influences the geographical distribution and seasonal fluctuations of malaria, but the effects are non-linear [8,15]. *Anopheles* mosquitoes thrive when the humidity level is at least 60%, which allows them to survive long enough to acquire and transmit malaria parasites [16,17]. High altitudes also constrain malaria transmission due to low temperatures and surface redistribution of water [18]. Crop cover and land use which includes deforestation and irrigation are also associated with expansion of mosquito larval habitats and may prolong the transmission season [19,20]. Despite the general relationship between rainfall, temperature, and humidity and mosquito abundance and malaria transmission, several studies have reported conflicting results regarding the effects of climatic and environmental factors on malaria transmission. These differences can be attributed to a wide range of modeling approaches, which may fail to fully capture the complex nonlinear relationships between climatic factors and malaria incidence, or may overlook important parameters in statistical regression models [21,22].

Although climatic and environmental factors directly impact malaria transmission, the ultimate effect of climatic variables remains unclear because other factors, such as socio-economic development and malaria control interventions, also play a role in transmission dynamics. Over the past 20 years, global campaigns have accelerated the scale-up of vector control interventions and case management with artemisinin-based combination therapies (ACTs), leading to a significant decline in malaria morbidity and mortality in endemic countries between 2000 and 2015 [23]. To produce accurate malaria forecasts, however, it is necessary to consider the joint effects of climatic and non-climatic factors. However, this is often not feasible due to lack of good quality data for quantifying these factors. The health and demographic surveillance systems (HDSS) in Africa offer unique opportunities to investigate the effects of both climate variability and malaria interventions. Nyawanda *et al.* demonstrated using a statistical model that in the lowlands of western Kenya, temperature, rainfall, and bed net use were significant factors influencing malaria transmission between 2008 and 2019 [24]. Using a nonlinear stochastic transmission model, Beloconi *et al.* further showed that additional contextual information regarding general population characteristics (such as in- and out-migration and the number of births and deaths) can enhance the understanding of the factors affecting malaria transmission thus improving the estimation and forecasting of malaria incidence [25]. However, the transmission model used malaria data aggregated across several villages and did not consider the impact of spatial heterogeneity on malaria transmission. To effectively model malaria transmission and implement intervention strategies, it is important to study multiple sub-populations concurrently [26]. Human transmission dynamics and intervention strategies are deeply embedded in the local ecology and conditions, making it unlikely that the same responses will apply universally. Additionally, movement of both humans and mosquitoes may lead to reintroduction and resurgence of parasites in low transmission areas [27].

A number of mathematical models have been developed to describe human migration, short-term movement between spatially heterogeneous regions, and their effects on malaria transmission. These models build on the Ross-Macdonald framework to achieve this goal [4,28–31]. More broadly, metapopulation models for malaria typically assume mosquito migration [32–35], necessitating entomological analysis. However, climatic factors were not incorporated into host metapopulation models for a considerable period of time [36]. Recently, variations in climate have been included in a deterministic malaria model to parameterise the basic reproductive number [37]. Other studies have examined how climate variability affects the sporogonic cycle of the parasite [27,38] or the mosquito life cycle when modelling the vector [34,39]. However, all of these methods require either additional movement data for both the host and the vector or further analysis to approximate mobility. Metapopulation models fitted to data using formulations similar to those in [25] can be promising in forecasting malaria dynamics by accounting for seasonal and spatial heterogeneity, and the confounding effects of different malaria drivers. While each of these frameworks addresses specific components of malaria transmission, there remains a need for models that jointly incorporate climatic variability, intervention coverage, host immunity, and spatial connectivity within a single mechanistic system.

In this study, we addressed this need by developing a nonlinear metapopulation stochastic transmission model that incorporates key mechanisms related to malaria dynamics, such as immunity and infectivity, as well as climatic factors and malaria control interventions at the sub-regional level, while accounting for spatial interactions between the host and the vector. This model unifies previously disjoint elements, such as climate sensitivity, spatial heterogeneity, intervention dynamics, and population immunity, into a single forecasting framework. By capturing these interacting mechanisms in a spatially structured system, it enables a more comprehensive understanding of malaria dynamics across diverse ecological settings. Although implemented with data from Kenya, the model is designed to be generalizable to other endemic regions with appropriate surveillance and demographic inputs.

## Results

### Overview of study area and methods

**Fig 1:**
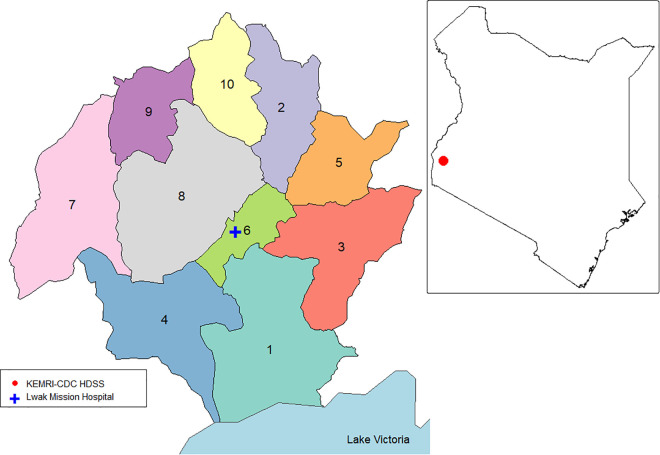
Study area. Location of the population-based infectious disease surveillance (PBIDS) area in western Kenya. The inset (top right) shows the position of the PBIDS study area within Kenya (red dot). The main panel displays the 10 clusters (IDs 1–10) used for analysis and the location of Lwak Mission Hospital within the study area. These maps were created using the tmap-package in R, and the basemap shapefiles were downloaded from ESPEN (2022) (https://espen.afro.who.int/maps-data/data-query-tools/cartography-database and https://data.humdata.org/dataset/cod-ab-ken).

The PBIDS study area is located next to Lake Victoria in Western Kenya and covers 33 rural villages situated within a 5 km radius from the St. Elizabeth Lwak Mission Hospital (LMH) (Fig 1). A distance-based hierarchical algorithm was used to group the 33 villages into 10 clusters [40], as depicted in Fig 1. These 10 clusters served as the common geographical unit for data aggregation in all subsequent analyses. S6 Fig maps the mean population per cluster over the whole study perioed, highlighting a marked heterogeneity (2,000–9,000 residents per cluster). The study area is relatively uniform in elevation, with village-level altitudes ranging from 1154 to 1315 meters above sea level, and cluster-level means ranging from 1172 to 1306 meters.

**Fig 2:**
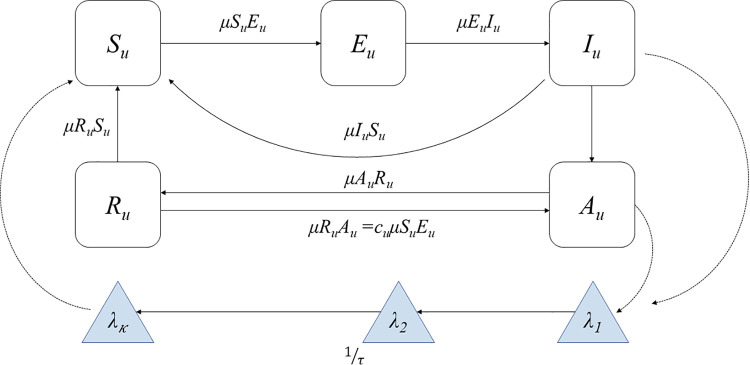
Diagram of the compartmental structure of the model. For each cluster *u*, the human population is divided into five classes: $S_{u}$- susceptible to infection; $E_{u}$ - exposed to parasites that have not yet matured into gametocytes; $I_{u}$ - infected with symptoms and infectious; $A_{u}$ - asymptomatic with reduced parasitemia; and $R_{u}$ - recovered and protected from severe infection. Transitions between the human compartments are denoted by a solid arrow with rate μ, and transitions between the human and mosquito compartments are denoted by dotted lines. The chain of compartments $\left(\lambda_{1},\cdots ,\lambda_{k}\right)$ implements a distributed time delay between infections in humans and the force of infection (the per-capita rate of infection) experienced by a susceptible individual, as described in Methods Section. The model is formalized by the stochastic differential equations (3)-(7).

The models were fitted to the time series of monthly malaria incidence from January 2008 to December 2019, and the estimated parameters were used to forecast malaria cases between January 2020 and December 2022. A sequential plug-and-play Monte Carlo approach [41,42] was used for likelihood maximization via iterated Block Particle Filtering (BPF) [43]. The flow diagram for one cluster is shown in Fig 2 and the stochastic differential equations in “Materials and methods” section This method performs sequential Monte Carlo while adding noise to perturb the parameters at each time point, thereby approximating the maximum likelihood estimates (MLE). An iterated BPF direct approach can achieve the MLE when the parameters are localized to a specific unit, i.e., when all parameters are unit-specific. Certain malaria transmission parameters, such as the fraction of asymptomatic individuals contributing to the force of infection (*q*) and the transition rates between compartments (*μ*), were expected not to vary substantially across subpopulations and were therefore assumed to be shared across all spatial units. Thus, out of a total of 21 parameters per cluster, 15 were assumed to be shared (S8 Table), while the remaining parameters were unit-specific. In particular, all the parameters associated with the transmission rates, $\mu_{E_{u}I_{u}}$, $\mu_{I_{u}S_{u}}$, $\mu_{I_{u}A_{u}}$, $\mu_{A_{u}CI_{u}}$, $\mu_{CI_{u}S_{u}}$, $q_{u}$, $c_{u}$, and *ψ*, were considered shared between units. The Akaike information criterion (AIC) [44], along with the mean absolute error (MAE) and root mean square error (RMSE) between observed and simulated cases, were used to compare models with and without climatic and seasonal terms and to evaluate their added value. In addition, we compared the coupled spatiotemporal model with a set of uncoupled temporal models fitted independently to each spatial unit to assess the contribution of spatial interactions. This comparison was based on the log-likelihoods, AIC, MAE, and RMSE. For the temporal only models, these values were summed under the assumption of no spatial coupling.

### Effects of climate and bed net use on malaria incidence

The trace plots of the parameters $\beta_{T}$, $\beta_{R}$, and $\beta_{I}$ corresponding to the effects of temperature, rainfall, and bed net use on malaria transmission, respectively, and the corresponding log-likelihood values during 250 iterations of the iterated block particle filtering algorithm, indicated convergence after aproximately 50 iterations (S1-S3 Figs). During the final 100 iterations, fluctuations in the estimated values were minimal, and the log-likelihood showed almost no change.

The coefficients of temperature ($\beta_{T}$) and bed net use ($\beta_{I}$) maximized the likelihood in the negative range of values (S1 and S3 Fig) for all clusters, suggesting a negative effect of temperature and bed net use on malaria transmission across the entire study area. The coefficients of rainfall appeared to converge around zero for spatial clusters 1 and 2, in the negative range for cluster 3, and in the positive range for all the other clusters (S2 Fig). This indicates cluster-specific shifts in the effecs of rainfall on transmission, with positive effects being predominant. To validate this result, purely temporal transmission models, similar to those in [25], were additionally fitted to clusters 1–4, and the results were consistent. Furthermore, S7 Fig shows a clear annual cycle in malaria cases that aligns with the rainy seasons, with case peaks lagging rainfall by 2 months. At the cluster level, S8 Fig shows broadly synchronous timing but varying amplitudes, indicating spatial heterogeneity in burden despite similar rainfall patterns.

The model that included splines, rainfall, temperature, and bed net use variables in the force of infection produced the best fit in terms of all three metrics (AIC: 12250.41, MAE: 140.2188, RMSE: 195.808). The model with only splines performed worse (AIC: 12260.42.5, MAE: 150.48, RMSE: 207.34), while the model without any covariates had the worst fit (AIC: 12762.84, MAE: 249.3403, RMSE: 335.4518) (S10 Table). In addition, a set of uncoupled temporal models fitted independently to each spatial unit resulted in higher overall error and model cost (AIC: 12350.58; MAE: 161.25; RMSE: 219.88), further supporting the added predictive value of the coupled spatiotemporal framework (S10 Table).

To further assess the added value of spatial coupling in our spatio-temporal model, we compared the full coupled model to the independent temporal models fitted separately to each spatial unit. Under the assumption of no interaction between units, the total log-likelihood of the uncoupled model was obtained by summing the log-likelihoods of the individually fitted regional models. We then used the Akaike Information Criterion (AIC) to compare model fit between the coupled and uncoupled approaches. As shown in S10 Table, the coupled model consistently achieved a lower AIC, indicating a better trade-off between fit and complexity. This quantitatively supports the importance of spatial interactions in capturing local transmission dynamics and improving fit beyond what isolated temporal models can provide.

### Estimated malaria transmission dynamics

The estimates of the best-fitted model, together with the ranges of the starting parameter values assumed to be shared across spatial clusters, are presented in S8 Table. Specifically, the best model suggests that the average time for moving from the exposed compartment (*E*) to the infected one (*I*) is 27.7 days, while the time it takes to return from the infected status (*I*) back to the susceptible status (*S*) without developing malaria immunity is 22.1 days. As expected, longer durations were estimated for the development of immunity (transitions from *A* to *R*) and subsequent loss of immunity (from *R* to *S*), with mean values of 453.2 and 216.4 days, respectively. The coefficient of reinfection with clinical immunity was estimated to be around 15% (c=0.15), and the relative infectivity of partially immune individuals was estimated at 84% (q=0.84).

**Fig 3:**
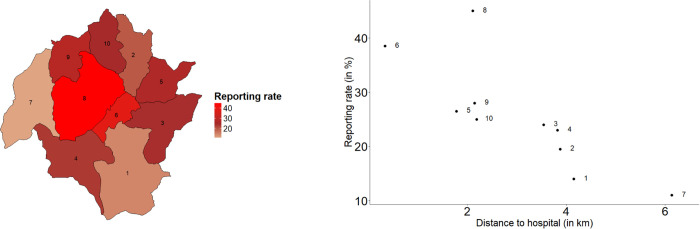
Reporting rates (in %) per cluster I-X. Estimated reporting rates ($\rho_{u}$) from the best-fitting model. Left: spatial distribution of reporting rates across clusters. Right: relationship between cluster distance to Lwak Mission Hospital and reporting rate. The base map is derived from population-based infectious disease surveillance (PBIDS) shapefiles provided by the Kenya Medical Research Institute (KEMRI). The map was generated in R (version 4.4.2) using the ggplot2 package.

The reporting rate $\rho_{u}$, which represents the percentage of symptomatic individuals who sought care at the hospital, was estimated separately for each cluster (S2 Table). Clusters 1 and 7, located farther from Lwak Mission Hospital, had the lowest reporting rates, while clusters 6 and 8, located closer, had the highest (Fig 3). The figure also shows the relationship between reporting rate and distance from the hospital.

The estimated initial values of the fraction of people in each of the five compartments and for each spatial cluster are shown in S3-S7 Tables. To determine an appropriate value for the spatial coupling parameter g, we evaluated the profile likelihood over the interval 0–100. The likelihood increased monotonically and achieved its maximum at the upper boundary of the tested range. Consequently, we fixed g=100 in the final model. The full profile likelihood curve is presented in S10 Fig.

### Model fit

**Fig 4:**
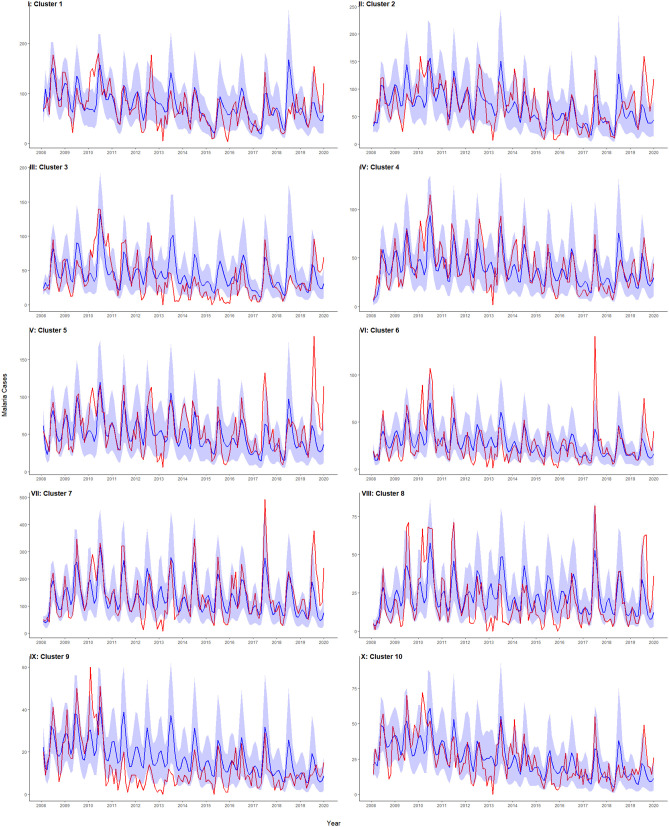
Model fit per cluster I-X. Observed monthly malaria cases are shown in red. The median of 1000 simulations from the best-fitting model is shown in blue, with prediction uncertainty represented by the 10 - 90% quantiles (shaded blue).

The best model (with MLE parameters) was used to generate 1000 simulations of malaria incidence from 2008 to 2019, based on estimated starting conditions in each cluster. Fig 4 depicts the observed monthly malaria incidence (in red) and the median of the simulated malaria cases (in blue), along with the prediction uncertainty, represented by the 10% and 90% quantiles (shaded in blue). The model adequately reproduced average transmission patterns across clusters. S9 Table reports the mean absolute error (MAE) and root mean square error (RMSE) between the median of these simulations and the observed malaria incidence per cluster. MAE ranged from 140.2 to 148.7 cases, while RMSE ranged from 195.5 to 209.6. chKenyametasimtotal shows the aggregated results across all 33 villages in PBIDS.

### Forecasting malaria incidence

**Fig 5:**
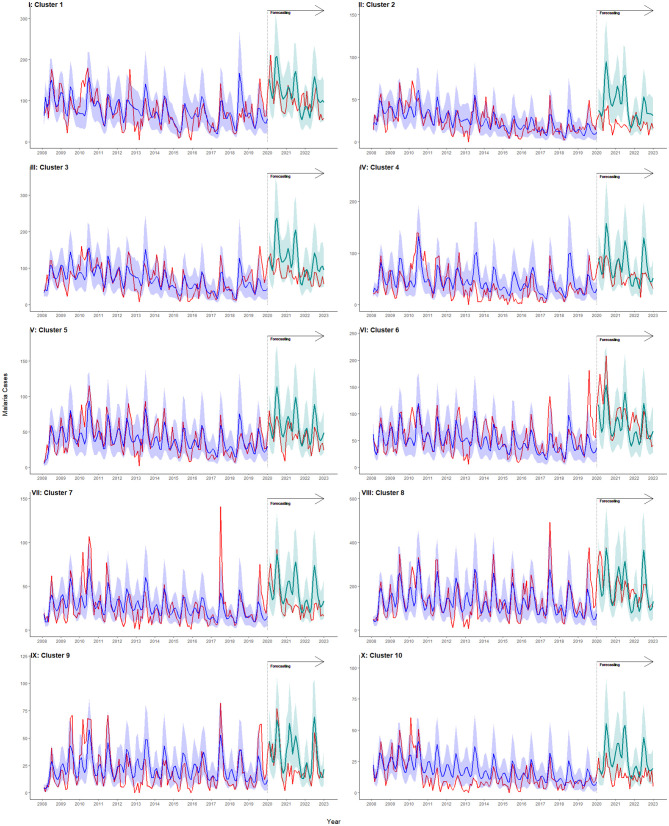
Malaria forecasts per cluster I-X. Observed monthly malaria cases are shown in red. The median of 1000 simulations from the best-fitting model is shown in blue, with prediction uncertainty (10 - 90% quantiles) shaded blue. Forecasted incidence for 2020 - 2022 is shown in cyan, with forecast uncertainty (10 - 90% quantiles) shaded in cyan.

Fig 5 compares observed (in red) and simulated (in blue) malaria cases during the training period (2008–2019) and observed with forecasted median incidence (in cyan) monthly for 2020–2022, based on 1000 simulations, including forecasting uncertainty (10–90% quantiles, shaded cyan). Overall, the model forecasted incidence reasonably well, though a slight overestimation was observed across most clusters. S5 Fig shows aggregated forecasts across the study region. Rainfall during the prediction period (2020–2022) was higher than in previous years, while mean land surface temperatures were modestly lower (S9 Fig). These climatic anomalies are consistent with environmental conditions that increase malaria transmission potential and likely contributed to the higher observed epidemic intensity during this period. In contrast, discrepancies between predicted and observed case counts during this period are discussed in terms of reporting and surveillance disruptions associated with the COVID-19 pandemic.

## Discussion

Understanding localized factors driving malaria transmission is essential for effective monitoring, evaluation, and accurate forecasting of malaria incidence, particularly in the context of climate change. Simpler malaria models that assume homogeneous populations have made significant contributions to malaria research over the last few decades. Nonetheless, the complexity of the disease necessitates the incorporation of spatial heterogeneity to facilitate a deeper understanding of effective malaria control strategies and account for the impact of small-scale factors related to malaria transmission. In this study, a spatio-temporal stochastic transmission model was developed that estimates the movement of both the vector and the host while quantifying the impact of neighboring regions on the force of infection. Furthermore, the model quantifies the effects of climate variability and malaria interventions at the local scale, as well as the small-scale heterogeneity in parameters related to population malaria dynamics.

Previous research has shown that temperature and precipitation significantly affect the growth and survival of both *P. falciparum* and mosquitoes [45]. Our model indicates that an increase in daytime land surface temperature with a one-month lag significantly reduced malaria incidence across all clusters. This consistent effect suggests that temperature acts in a largely monotonic fashion in this setting. Similar temperature-related reductions in malaria incidence have been reported in endemic areas of Kenya and Uganda [8,25,46,47]. The *Anopheles*s mosquito population, which transmits malaria, declines sharply at temperatures above 35 ^∘^C [48], and our observed temperature data show that clusters in this study frequently exceed this threshold. This relationship aligns with the known non-linear suitability curve for malaria transmission, which peaks at 25–27^∘^C and decreases at higher temperatures [49].

Rainfall with a two-month lag had a positive effect on malaria incidence in most of the spatial units. These findings are consistent with our earlier analyses in this study area, which reported a significant positive effect of rainfall on malaria transmission in models fitted to aggregated data from all 33 villages [24,25]. However, in this analysis, two clusters (1 and 3) revealed no significant effect of rainfall, and one cluster even showed a negative effect. These disparities may reflect additional dynamics and geographical heterogeneity, which the current metapopulation model could better capture by using spatially disaggregated data. Specifically, clusters located farther from the main hospital (Lwak Mission Hospital), face more difficult access during heavy rains. Adults with less severe symptoms may therefore seek treatment at other medical facilities or pharmacies. Furthermore, because the relationship between rainfall and malaria is non-linear, these findings may also suggest that heavy rains in these clusters washed away mosquito breeding sites [50].

Earlier studies have reported a strong link between malaria risks and non-climatic factors such as malaria interventions, socio-economic development, and geographical setting [51]. The main malaria interventions in Kenya include bed net distribution, indoor residual spraying (IRS), and prompt diagnosis and treatment with ACTs [52]. In our study, only bed net use was considered in the model because IRS was not conducted in the study area during these years and all malaria cases were treated with ACT. Bed nets are widely used in rural communities in western Kenya [53]. Our model revealed that increased bed net use had a significant negative effect on the force of infection and, consequently, on malaria incidence across the study area. Despite widespread use, malaria cases have increased since 2016. This may be explained by climatic changes during this period and a shift in mosquito species composition and behaviour [54,55]. Incorporating both climate and intervention data improved model fit, as reflected by lower AIC, MAE, and RMSE metrics compared to simpler models that do not account for these factors.

The proportion of severe malaria cases was relatively low in the study area, and the majority of reported infections at Lwak Mission Hospital were in children [56]. Our model estimated that the average reporting rate per cluster was about 25%. Furthermore, a clear inverse relationship was identified between the distance of a cluster of villages from the main hospital and the estimated reporting rate in each sub-region. This supports the assumption that people prefer to visit nearby medical facilities or purchase drugs over the counter, especially during the rainy season when travel to the hospital is more difficult.

Our model estimated that the first symptoms of *P. falciparum* malaria appear on average 27.7 days after infection. This estimate is slightly higher than the WHO range (10–15 days) [7], but consistent with the broader U.S CDC range (7–30 days) (https://www.cdc.gov/malaria/about/disease.html). The longer delay may reflect additional time between symptom onset and hospital reporting, or the role of acquired immunity in the population [57,58]. Since all age groups were modelled together, the contribution of immune individuals is significant, as reflected in our estimates showing a large recovered afraction ($R_{u}[t]$) across clusters throughout the study period.

The model demonstrated good forecasting ability for malaria incidence in 2020–2022 across all clusters. However, forecasts slightly overestimated incidence. This likely reflects higher rainfall and lower temperatures during 2020–2022 [59], both of which increase transmission in the model [24]. The COVID-19 pandemic may also have influenced care-seeking, lowering observed case counts. A recent study in the area reported reduced hospital reporting during the pandemic [60], potentially explaining the observed overestimation.

Importantly, overestimation varied across clusters. In some areas, the discrepancies were larger, likely reflecting spatial heterogeneity in both COVID-19-related intervention disruptions [61] and healthcare access. Clusters located farther from health facilities may have experienced sharper declines in reporting, reducing observed incidence, while others were less affected. This underscores the importance of localized contextual factors in shaping disease dynamics, and highlights the need for models that can incorporate real-time changes in intervention coverage and health-seeking behavior across space.

The anomaly observed in 2013 reflects contextual rather than biological factors. The March 2013 general elections, the first held after the 2007–2008 post-election violence, may have influenced care-seeking behavior, with households stockpiling antimalarials and deferring visits to health facilities [62]. In addition, nationwide health worker strikes in late 2013 disrupted services reducing facility attendance [63]. In Cluster 9, the model fit is poorer because it projects a continuation of the previous trend, whereas the observed data show a sharp decline. This was likely due the opening of new health facilities, including Rambugu and later Bar Aluru dispensaries, which diverted patient attendance away from LMH [64].

A major computational challenge of this analysis was the multimodal likelihood function with many unknown parameters. This is particularly problematic when parameter combinations are weakly identifiable. Allowing certain transmissio parameters to vary across time and space can lead to over-parameterization, as each additional component requires estimating more parameters. Some potentially important variables were excluded because they lacked temporal or spatial variation. For example, crop cover and surface water percentages were tested but showed little variability and failed to converge. Relative humidity and wind speed also exhibited minimal spatial variation and were excluded. The spatial coupling parameter *g*, in the gravity model was fixed at 100 after exploratory testing, as yielded the best log-likelihood. Attempts to estimate *g* directly produced unstable behavior and poor fits, likely due to limited spatial resolution and identifiability constraints. Future work could consider alternative estimation strategies, such as profile likelihood or hierarchical modeling approaches. Another limitation is the assumption of a homogeneous host population. Malaria risk, immunity development, and mortality vary by age, with children under five bearing the highest burden. Future studies should consider explicitly modeling age structure using age-stratified frameworks.

When modelling malaria dynamics, it is important to include parameters that account for the total flux of individuals between different locations. Although our study focused on a relatively small area with modest climatic variation across clusters, the spatio-temporal model captured key mechanisms of malaria transmission, such as seasonal disease fluctuations, and more accurately estimated local-scale heterogeneity within sub-populations than a purely dynamic model applied to aggregated data [25]. The model is therefore a powerful planning tool for forecasting malaria incidence at different geographical scales under various intervention scenarios, while accounting for climatic conditions, mobility, and localised contextual factors.

## Materials and methods

### Ethics statement

The HDSS and PBIDS study protocols were reviewed and approved by the Kenya Medical Research Institute (KEMRI) Scientific and Ethics Review Unit (SERU # 1801 and 2761) and Center of Disease and Prevention’s (CDC) Institutional Review Board (CDC IRB # 3308 and 6775). All patients, or their parents or legal guardians if they were minors, provided written informed consent. In addition, compound heads provided written informed consent for the household-based evaluation.

### Data sources

The Kenya Medical Research Institute (KEMRI), in collaboration with the US Centers for Disease Control and Prevention (CDC), has conducted population-based infectious disease surveillance (PBIDS) since 2005 in Asembo, Siaya County [65]. PBIDS is embedded within the health and demographic surveillance system (HDSS) and covers approximately 35,000 people (as of 2024) residing in 33 rural villages within a 5 km radius of St. Elizabeth Lwak Mission Hospital (LMH), located near Lake Victoria (Fig 1). KEMRI, with support from CDC, routinely collects data on malaria incidence, control interventions, vector densities, migration, and household-related indicators in western Kenya [65,66]. Births, deaths, and migration flows used in the model are also derived directly from the HDSS-recorded demographic events, and population sizes are dynamically updated based on these data rather than imposed through fixed rates or census counts. Population characteristics of the study area have been previously described [24,66].

### Malaria incidence data

In this study, monthly malaria incidence data collected at LMH between January 2008 and December 2022 were analyzed. All individuals who visited the hospital with febrile illness were tested for malaria using blood smear microscopy. Every positive case was then treated with an artemisinin-based combination therapy (ACT), in accordance with Kenyan Ministry of Health guidelines. Children under the age of six months were excluded from the study because they are considered protected by maternal antibodies [67].

### Malaria intervention data

Thee proportion of individuals who reported using a bed net the night before the household interview was used as a measure of bed net coverage. Household visit data collected biweekly within the PBIDS area between January 2008 and April 2015 [65] were aggregated by month. After 2015, the frequency of household visits was reduced to two per year, but the data collection methods remained unchanged and the household visits were conducted systematically across different villages, organized into eight clusters within the HDSS.

### Climatic data

Daytime land surface temperature (LSTD) with a 1×1 $km^{2}$ spatial and an 8-day temporal resolution, was used as a proxy for air temperature. The data were obtained from the Moderate Resolution Imaging Spectroradiometer (MODIS) on board NASA’s Terra and Aqua satellites [68]. Rainfall data were obtained from the Climate Hazards Group InfraRED Precipitation with Station data (CHIRPS) at a 5.6×5.6 $km^{2}$ spatial and 5-day temporal resolution [69]. All climatic variables were aggregated to the monthly scale for each of the 10 clusters.

### Model formulation

**Fig 6:**
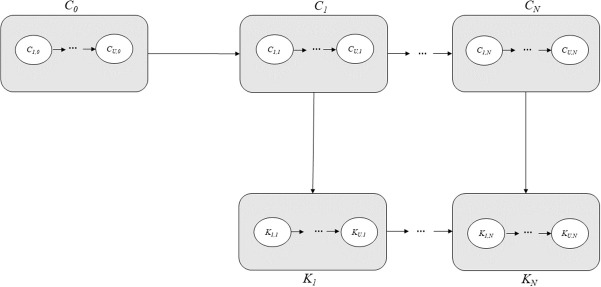
Metapopulation model. Diagrammatic representation of a spatio-temporal partially observed Markov process. The value of the latent process at time $t_{n}$ is denoted by $C(t_{n})=(C_{1,n},\cdots ,C_{U,n})$, and the partial and noisy observations are modeled by $K(t_{n})=(K_{1,n},\cdots ,K_{U,n})$, where there are *U* units labeled 1:U={1,⋯,U}.

A metapopulation stochastic transmission model for *P. falciparum* dynamics was formulated based on previous modifications of the susceptible-exposed-infected-recovered (SEIR) model and implemented within the framework of a partially observed Markov process (POMP). Specifically, the model compartmentalizes the population of each cluster into susceptible (S), exposed (E), infectious (I), asymptomatic with reduced parasitemia (A), and recovered and protected from severe infection (R) [25]. Fig 6 depicts the diagram of the spatio-temporal partially observed Markov process (SpatPOMP) employed in this work [70]. The compartmental structure of the model for one spatial unit (i.e., each cluster) is illustrated in Fig 2.

A notation for the metapopulation SpatPOMP model was established, extending the POMP notation of [25]. Suppose there are *U* spatial units, u∈{1,⋯,U}, and that $\mu_{X_{u}Y_{u}}$ denotes the rate of transmission from compartment $X_{u}\text{ to }Y_{u}$, $X_{u}$, $Y_{u}\in \{S_{u}(t),E_{u}(t),I_{u}(t),A_{u}(t),R_{u}(t\}$ in each unit. The model considers the possibility of individuals that have low or no protective immunity after infection by transitioning directly from the initial infectious state ($I_{u}$) back to susceptibility ($S_{u}$), bypassing those asymptomatic with reduced parasitemia ($A_{u}$) and those completely recovered ($R_{u}$). This pathway is particularly relevant for children who may not yet have acquired immunity, or adults who may have lost immunity. Additionally, a relationship is assumed between the rates of transition from asymptomatic to recovered, $\mu_{A_{u}R_{u}}=c_{u}\mu_{S_{u}E_{u}}$, characterized by a constant of proportionality $c_{u}$, with $0\leq c_{u}\leq 1$.

A time-delayed equation was employed to describe the force of infection $\mu_{S_{u}E_{u}}$, incorporating both parasite development within the mosquito and the survival probability of the mosquito up to the point of transmission [71,72].

At time *t* and unit *u* the force of infection is defined as:


$$\mu_{S_{u}E_{u}}=\int_{-\infty }^{t}\gamma_{u}(t-s)\lambda_{u}(s)d\Gamma_{u}(s),$$ (1)

where $\gamma_{u}(t-s)$ is a Gamma distribution, representing the delay of the parasite life cycle inside the vector and its survival, specifically $Gamma(k,\frac{\tau_{u}^{2}}{m})$:


$$\gamma_{u}(t)=\frac{(m/\tau_{u}{)}^{m}t^{m-1}}{(m-1)!}\exp\{-mt/\tau_{u}\},$$ (2)

and λ denotes the force of infection at a previous time *s* when a mosquito bites an infected human.

Additionally, for each unit *u*, the number of births ($B_{u}(t$), deaths ($D_{u}(t$), and in- and out-migration ($MIN_{u}(t)$ and $MOUT_{u}(t)$) were included. Newborns were assumed to enter the susceptible compartment six months after birth. Migration was assumed to occur at equal rates in and out of each compartment within each unit, and the death rate was assumed equal across all compartments. Lastly, $P_{u}(t)$ denotes the total population of unit *u* at time *t*. The following stochastic differential equations formalize the transmission structure within each unit:


$$\begin{array}{ccc} & & \frac{dS_{u}}{dt}=B_{u}S_{u}+MIN_{u}S_{u}+\mu_{R_{u}S_{u}}[u]R_{u}+\mu_{I_{u}S_{u}}[u]I_{u}-\mu_{S_{u}E_{u}}[u]S_{u}\\ & & -D_{u}S_{u}-MOUT_{u}S_{u},\; \end{array}$$ (3)

$$\frac{dE_{u}}{dt}=MIN_{u}E_{u}+\mu_{S_{u}E_{u}}[u]S_{u}-\mu_{E_{u}I_{u}}[u]E_{u}-D_{u}E_{u}-MOUTR_{u}E_{u},$$ (4)

$$\begin{array}{ccc} & & \frac{dI_{u}}{dt}=MIN_{u}I_{u}+\mu_{E_{u}I_{u}}[u]E_{u}-\mu_{I_{u}S_{u}}[u]I_{u}-\mu_{I_{u}I_{u}}[u]I_{u}-D_{u}I_{u}\\ & & -MOUT_{u}I_{u},\; \end{array}$$ (5)

$$\begin{array}{ccc} & & \frac{dA_{u}}{dt}=MIN_{u}A_{u}+\mu_{I_{u}A_{u}}[u]I_{u}-\mu_{A_{u}R_{u}}[u]A_{u}+\mu_{R_{u}A_{u}}[u]R_{u}-D_{u}A_{u}\\ & & -MOUT_{u}A_{u},\; \end{array}$$ (6)

$$\begin{array}{ccc} & & \frac{dR_{u}}{dt}=MIN_{u}R_{u}+\mu_{A_{u}R_{u}}[u]A_{u}-\mu_{R_{u}A_{u}}[u]R_{u}-\mu_{R_{u}S_{u}}[u]R_{u}\\ & & -D_{u}R_{u}-MOUT_{u}R_{u}.\; \end{array}$$ (7)

The latent force of infection in each unit is composed of local and spatial components:


$$\lambda_{u}(t)=\beta_{u}[y_{u}+\eta_{u}],$$ (8)

where $y_{u}(t)$ represents the proportion of infectious individuals within unit *u*:


$$y_{u}=\frac{I_{u}(t)+q[u]A_{u}(t)}{P_{u}(t)}.$$

This term captures the local infectious reservoir, where q[u]∈[0,1] is the relative contribution of asymptomatic individuals to onward transmission.

To allow transmission dynamics in each spatial unit to be influenced by those in neighboring units, we included a phenomenological coupling term $\eta_{u}(t)$ that smooths infectious pressure across space


$$\eta_{u}=\sum_{\bar{u},\bar{u}\neq u}\frac{k_{u\bar{u}}}{P_{u}(t)}(\frac{I_{\bar{u}}(t)+q[\bar{u}]A_{\bar{u}}(t)}{P_{\bar{u}}(t)}-\frac{I_{u}(t)+q[u]A_{u}(t)}{P_{u}(t)}).$$ (9)

The interaction weight $k_{u\bar{u}}$ determines the strength and spatial reach of this smoothing and is derived from a gravity kernel [73]


$$k_{u\bar{u}}=g\frac{P_{u}(t)P_{\bar{u}}(t)}{dist(u,\bar{u})}\times \frac{\overset{―}{dist}}{{\overset{―}{P}}^{2}}$$ (10)

where, $dist(u,\bar{u}$ is the distance between units *u* and $\bar{u}$; $\overset{―}{dist}$ is the average distance across unit pairs, and $\overset{―}{P}$ is the average population size. This formulation provides a diffusion-like approximation in which units with higher infectious pressure exert stronger epidemiological influence on neighboring units with lower pressure, and vice versa. It serves as an empirical means of borrowing information across units in the absence of explicit data on host mobility or vector dispersal. The scalar parameter g governs the strength of spatial coupling and was fixed at g=100 based on model performance after testing values in the range 0–100.

The transmission rate β[u] is defined as:


$$\beta [u]=\exp(\beta_{T}[u]\cdot LSTD_{u}+\beta_{R}[u]\cdot Rain_{u}+\beta_{I}[ucdotInt_{u}+\sum_{i=1}^{n_{s}}\beta_{i}[u]s_{u}^{i}(t))$$ (11)

where $\beta_{T}$, $\beta_{R}$, and $\beta_{I}$ correspond to the effects of daytime land surface temperature ($LSTD_{u}$), precipitation ($Rain_{u}$) and bed net use ($Int_{u}$), respectively. All covariates were standardized so that coefficients are dimensionless. Seasonality was modelled using the coefficients $\beta_{i}[u]$ of a periodic cubic B-spline basis $\left\{s_{i}^{u}(t),i=1,\cdots ,n_{s}\right\}$ with $n_{s}$ evenly spaced knots. Cumulative monthly rainfall with a two-month lag and daytime land surface temperature with a one-month lag were selected based on our earlier analyses [24]. Initial coefficient values were drawn uniformly from [-4,4], then refined to [-1,1] after examining convergence..

The latency in the force of infection was assumed *Gamma*-distributed with mean $\tau_{u}$ and variance $\tau_{u}/m$, to allow a differential representation that facilitates numerical solution. Considering *m* classes (for i=1,...,m), up to the current force of infection $\lambda_{m}$, the delay in the effect of λ on infection of human classes $S_{u}$ and $R_{u}$ was defined as:


$$\frac{d\lambda_{u}^{1}(t)}{dt}=(\lambda_{u}-\lambda_{u}^{1})m\tau_{u}^{-1}\frac{d\Gamma_{u}}{dt}$$ (12)

$$\frac{d\lambda_{u}^{2}(t)}{dt}=(\lambda_{u}^{1}-\lambda_{u}^{2})m\tau_{u}^{-1},$$ (13)

$$\frac{d\lambda_{u}^{i}(t)}{dt}=(\lambda_{u}^{i-1}-\lambda_{u}^{i})m\tau_{u}^{-1}\;\;\text{ for }i=3,...,m,$$ (14)

Here, $\tau_{u}$ is the *Plasmodium* development rate:


$$\tau_{u}=0.000111\cdot AirT_{u}\cdot (AirT_{u}-14.7)\cdot \sqrt{34.4-AirT_{u}},$$ (15)

where $AirT_{u}$ is the air temperature per unit [74]. By setting $\mu_{S_{u}E_{u}}=\lambda_{u}^{m}(t)$, the equations (12) - (14) are equivalent to equation (2). $\Gamma_{u}(t)$ in equations (12) - (14) denotes a *Gamma* process representing integrated noise with intensity $\sigma^{2}$ and stationary independent increments such that $\Gamma_{u}(t)-\Gamma_{u}(s)\sim Gamma([t-s]/\sigma_{u}^{2},\sigma_{u}^{2}$. The *Gamma* process is chosen instead of *Gaussian* noise, to enforce the positivity of $\mu_{S_{u}E_{u}}$ and all the state variables in equations (3) - (8). After investigation, a total of m=2 classes was selected. The system was solved numerically via the Euler method [75] with a time step of 1 day.

The new infections $H_{u}(n)$ that are sampled in the simulations of the transitions between the $E_{u}$ and $I_{u}$ compartments during the *n*-th interval are defined as:


$$H_{u}(n)=\int_{t_{n-1}}^{t_{n}}\mu_{E_{u}I_{u}}E_{u}(s)ds,$$

where $\left\{t_{n},n=1,...,N\right\}$ denotes the time of the observation, and the model is initialized at some time $t_{0}<t_{1}$. To account for potential under-reporting and measurement errors in the number of confirmed cases $y_{n}$, a measurement model characterized by a negative binomial distribution was assumed for each cluster, so that $y_{n}|H_{n}\sim NegBin(\rho H_{n},\psi^{2})$ with overdispersion parameter *ψ* and reporting rate *ρ*.

### Software

The methodology was implemented using the R package SpatPOMP [70]. A range of starting values was explored (S8 Table) to estimate the MLE parameters. The best-fitting models were then used to generate 1000 malaria simulations for each month, from which the median and 10–90% percentiles were reported. The code is openly available on Zenodo (DOI: https://doi.org/10.5281/zenodo.16994361).

## Supporting information

S1 FigEffects of land surface temperature per cluster I-X.Convergence of the $\beta_{T}$ parameters per cluster corresponding to the daytime land surface temperature. The plot illustrates the variation in the parameter estimates and the log-likelihood (loglik), plus a loglik zoomed y-axis view, over 250 iterations in the iterative block particle filtering algorithm. Starting values for all regression coefficients were drawn uniformly from the interval [-1,1], with only small variability remaining after approximately the 50th iteration.(TIFF)

S2 FigEffects of rainfall per cluster I-X.Convergence of the $\beta_{R}$ parameters per cluster, corresponding rainfall. The plot illustrates the variation in the parameter estimates and the log-likelihood (loglik), plus a loglik zoomed y-axis view, over 250 iterations in the iterative block particle filtering algorithm. Starting values for all regression coefficients were drawn uniformly from the interval [-1,1], with only small variability remaining after approximately the 50th iteration.(TIFF)

S3 FigEffects of bed net use per cluster I-X.Convergence of the $\beta_{I}$ parameters per cluster, corresponding to bed net use. The plot illustrates the variation in the parameter estimates and the log-likelihood (loglik), plus a loglik zoomed y-axis view, over 250 iterations of the iterative block particle filtering algorithm. Starting values for all regression coefficients were drawn uniformly from the interval [-1,1], with only small variability remaining after approximately the 50th iteration.(TIFF)

S4 Fig Model fit for the entire study area. Monthly malaria cases aggregated across the whole study area are depicted in red, and the median of 1000 simulations from the best model (with parameters that maximize the likelihood) is illustrated in blue, with prediction uncertainty (10–90% quantiles) shaded in blue.
(TIFF)

S5 Fig Malaria forecasts for the entire study area. Monthly malaria cases aggregated across the whole study area are depicted in red, and the median of 1000 simulations from the best model (with parameters that maximize the likelihood) is illustrated in blue, with prediction uncertainty (10–90% quantiles) shaded in blue. The median of the forecasted cases for 2020–2022 is shown in cyan, with forecast uncertainty shaded in cyan.
(TIFF)

S6 Fig Mean population per cluster. Map of the mean population per cluster during the study period (2008–2020) in the PBIDS surveillance area of western Kenya. The base map is derived from population-based infectious disease surveillance (PBIDS) shapefiles provided by the Kenya Medical Research Institute (KEMRI). The map was generated in R (version 4.4.2) using the ggplot2 package.
(TIFF)

S7 Fig Time series of observed malaria cases, scaled rainfall, and model-inferred seasonal forcing. Monthly malaria case counts (red), rainfall with a two-month lag (scaled, blue), and the seasonal component inferred from the model (scaled, green) from 2008–2020.
(TIFF)

S8 Fig Temporal dynamics of malaria Cases and rainfall by cluster. Time series of monthly observed malaria cases (black) and scaled rainfall (blue) for each of the 10 clusters, 2008–2020.
(TIFF)

S9 Fig Temporal dynamics of malaria cases, rainfall and temperature across the study period. Time series of monthly observed malaria cases (black), scaled rainfall (blue) and scaled temperature (red), 2008–2022.
(TIFF)

S10 Fig Profile likelihood curve of the gravity parameter *g.* Profile likelihood curve of the parameter *g*, which governs the strength of spatial coupling, and the corresponding interval (computed as the points at which the profile curve crosses the horizontal line five log-likelihood units below the maximum likelihood estimate).
(TIFF)

S1 Table Cluster identification numbers (Cluster ID) and village identification numbers (Village ID) used to identify the villages contained within each cluster. (PDF)

S2 Table Fitted reporting rate parameters ρ per cluster in the best malaria spatio-temporal stochastic transmission model. Starting values for all parameters were [0%, 100%].
(PDF)

S3 Table Fitted parameters of the susceptible compartment (*S*) per cluster in the best malaria spatio-temporal stochastic transmission model. Starting values for all parameters were [0%, 100%].
(PDF)

S4 Table Fitted parameters of the exposed compartment (*E*) per cluster in the best malaria spatio-temporal stochastic transmission model. Starting values for all parameters were [0%, 100%].
(PDF)

S5 Table Fitted parameters of the infectious compartment (*I*) per cluster in the best malaria spatio-temporal stochastic transmission model. Starting values for all parameters were [0%, 100%].
(PDF)

S6 Table Fitted parameters of the asymptomatic compartment with reduced parasitemia (*A*) per cluster in the best malaria spatio-temporal stochastic transmission model. Starting values for all parameters were [0%, 100%].
(PDF)

S7 Table Fitted parameters of the recovered and protected from severe infection compartment (*R*) per cluster in the best malaria spatio-temporal stochastic transmission model. Starting values for all parameters were [0%, 100%].
(PDF)

S8 Table Fitted parameters shared across clusters in the best malaria spatio-temporal stochastic transmission model. (PDF)

S9 Table Mean absolute error (MAE) and root mean square error (RMSE) between observed malaria cases and the median of 1000 simulated forecasts from the best model. (PDF)

S10 Table In-sample performance of models with different covariate sets in the force of infection. Metrics reported include the Akaike information criterion (AIC), mean absolute error (MAE), and root mean square error (RMSE). For the temporal model fitted separately to each region, metrics are summed across regions under the no-coupling assumption (log-likelihood, AIC, MAE, RMSE).
(PDF)
