# Supplementary figures and images for: Assessing the impact of climate and control interventions on spatio-temporal malaria dynamics using a stochastic metapopulation model
Source: PLoS Comput Biol. 2026 Mar 17;22(3):e1014004. doi: 10.1371/journal.pcbi.1014004 (PMC12995307; doi:10.1371/journal.pcbi.1014004)

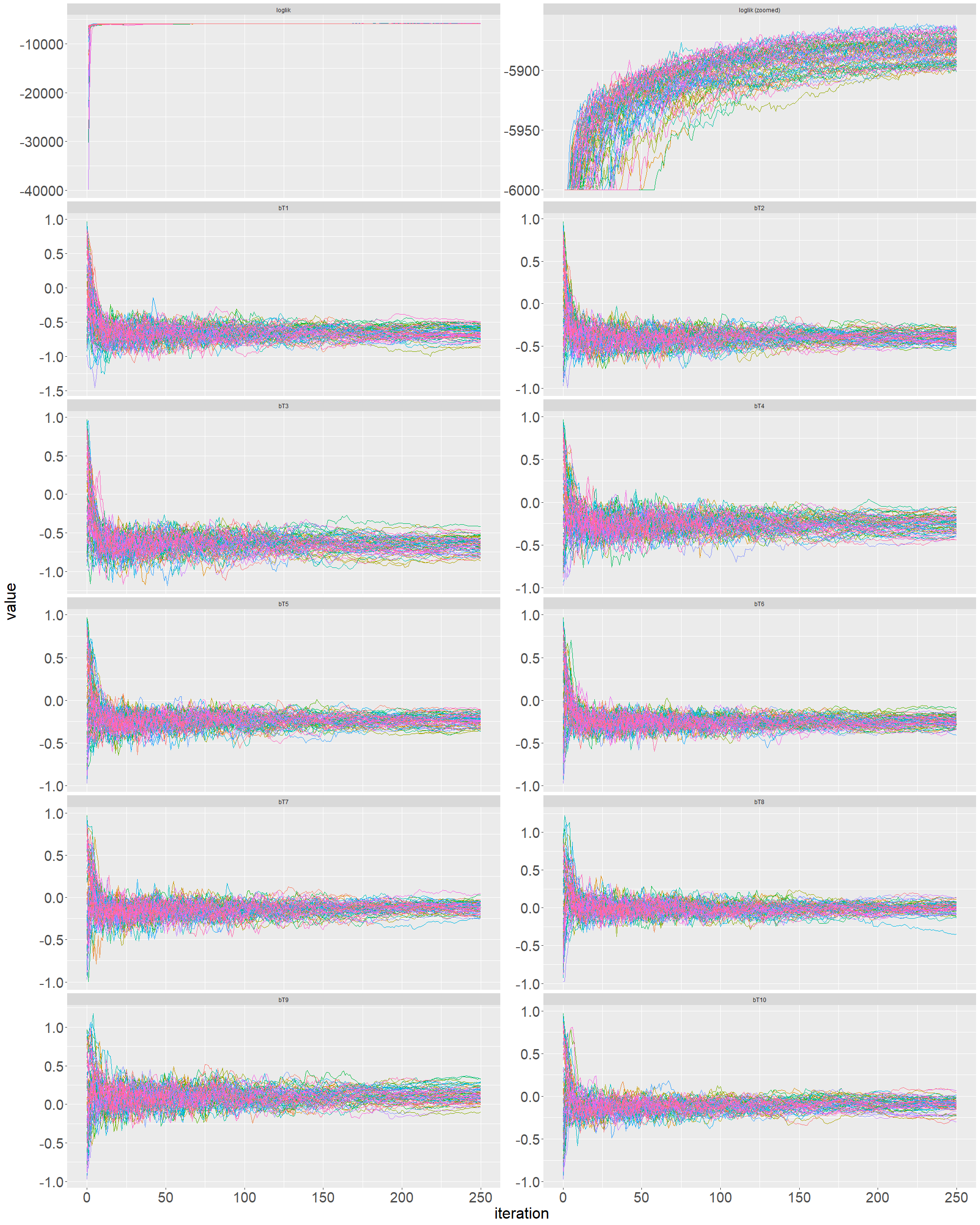

Supplement: S1 Fig — Convergence of the βT parameters per cluster corresponding to the daytime land surface temperature. The plot illustrates the variation in the parameter estimates and the log-likelihood (loglik), plus a loglik zoomed y-axis view, over 250 iterations in the iterative block particle filtering algorithm. Starting values for all regression coefficients were drawn uniformly from the interval [-1,1], with only small variability remaining after approximately the 50th iteration. (TIFF) [file pcbi.1014004.s001.tiff]

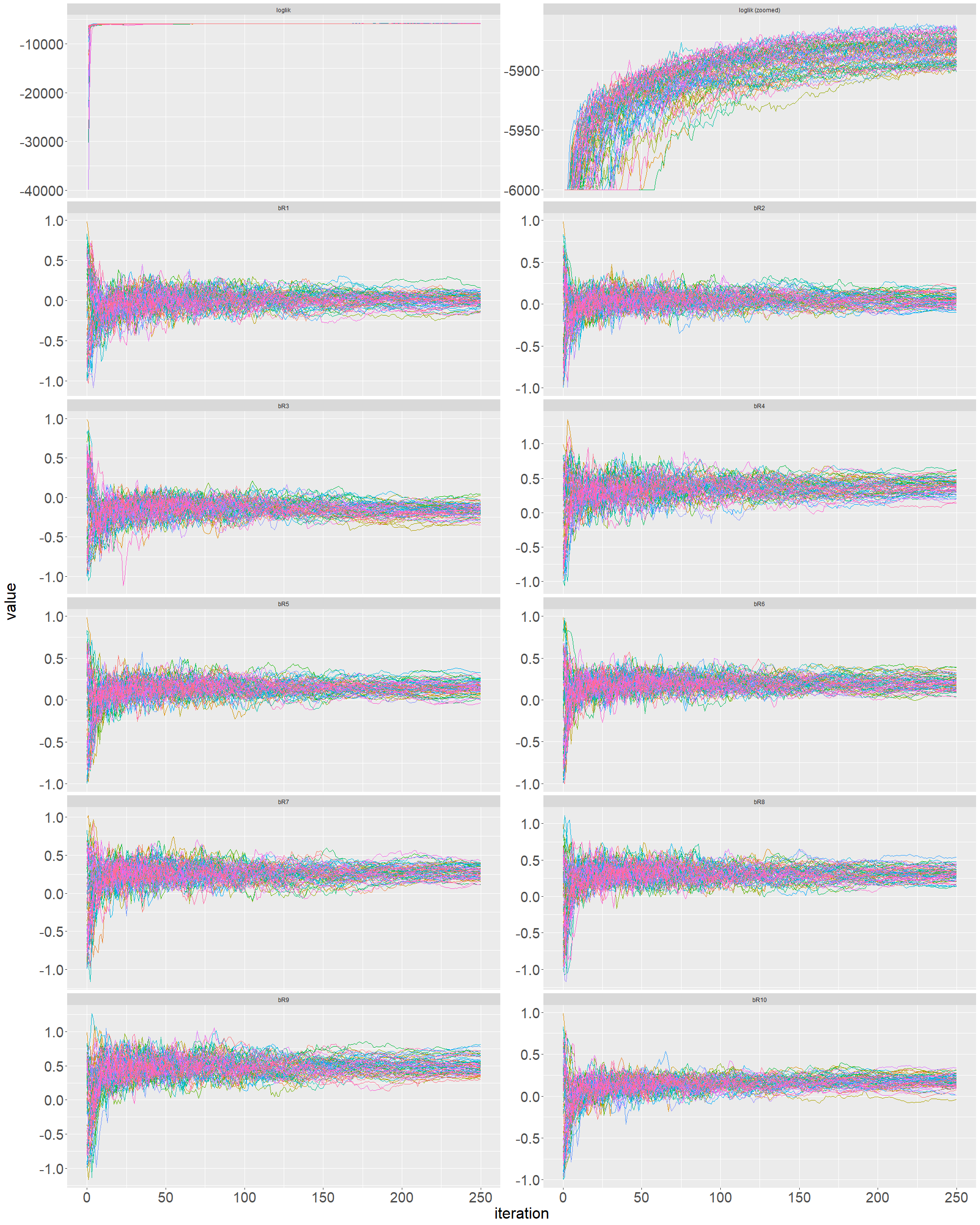

Supplement: S2 Fig — Convergence of the βR parameters per cluster, corresponding rainfall. The plot illustrates the variation in the parameter estimates and the log-likelihood (loglik), plus a loglik zoomed y-axis view, over 250 iterations in the iterative block particle filtering algorithm. Starting values for all regression coefficients were drawn uniformly from the interval [-1,1], with only small variability remaining after approximately the 50th iteration. (TIFF) [file pcbi.1014004.s002.tiff]

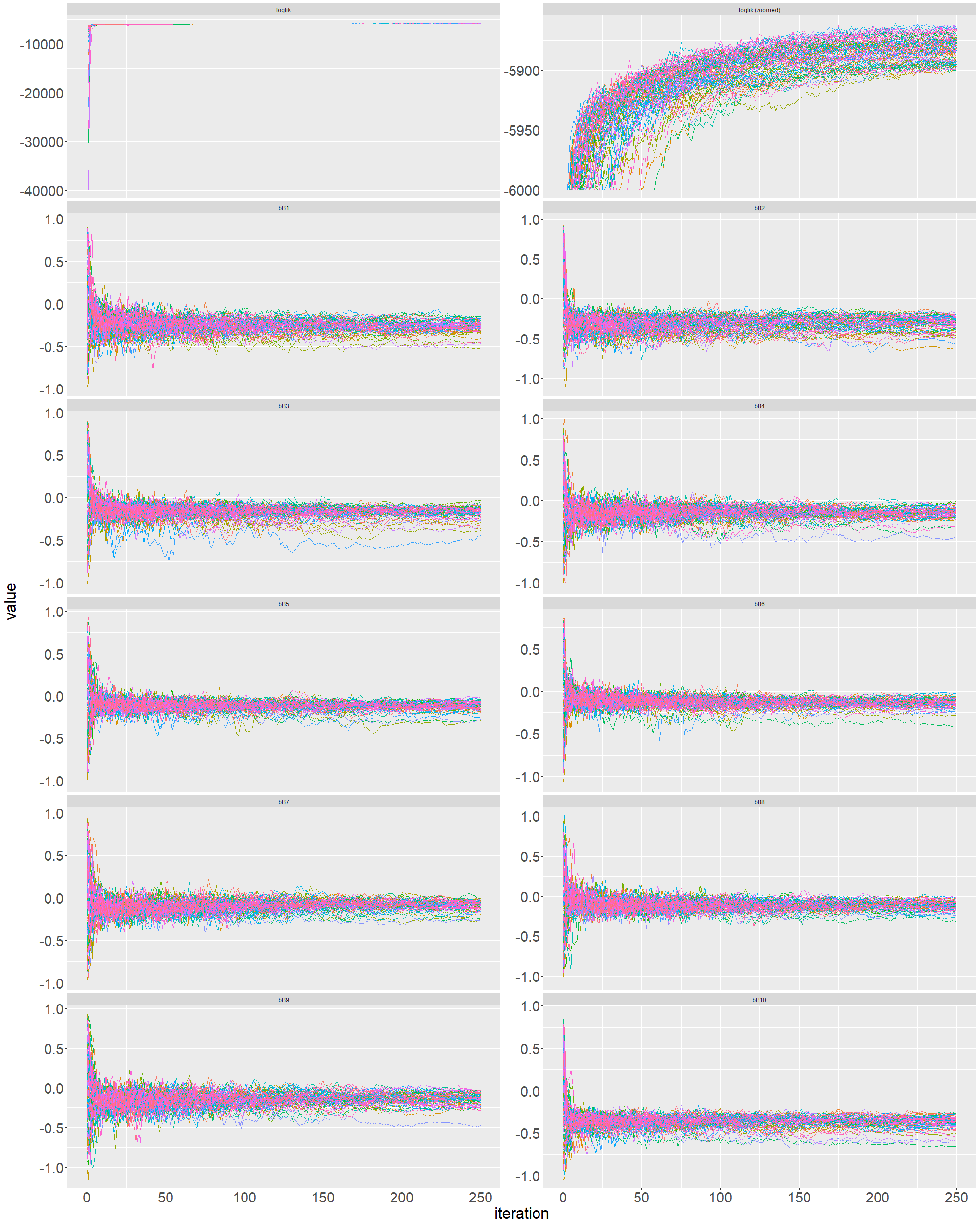

Supplement: S3 Fig — Convergence of the βI parameters per cluster, corresponding to bed net use. The plot illustrates the variation in the parameter estimates and the log-likelihood (loglik), plus a loglik zoomed y-axis view, over 250 iterations of the iterative block particle filtering algorithm. Starting values for all regression coefficients were drawn uniformly from the interval [-1,1], with only small variability remaining after approximately the 50th iteration. (TIFF) [file pcbi.1014004.s003.tiff]

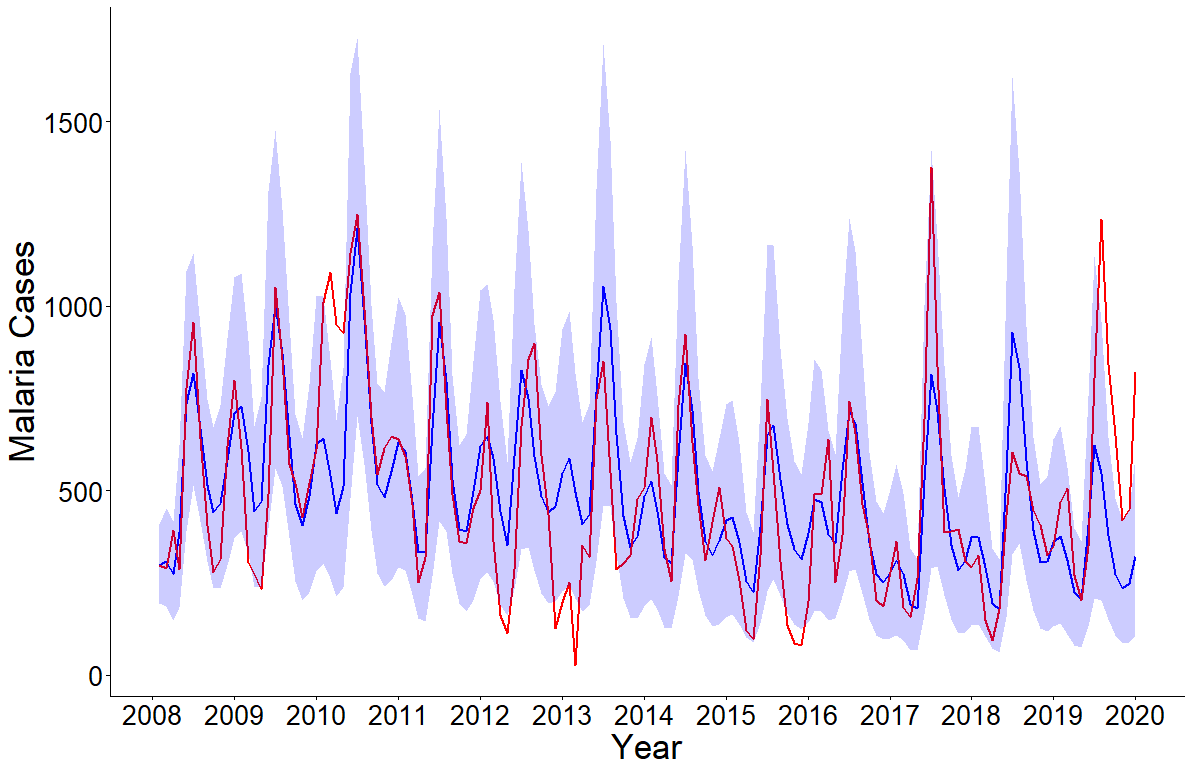

Supplement: S4 Fig — Monthly malaria cases aggregated across the whole study area are depicted in red, and the median of 1000 simulations from the best model (with parameters that maximize the likelihood) is illustrated in blue, with prediction uncertainty (10–90% quantiles) shaded in blue. (TIFF) [file pcbi.1014004.s004.tiff]

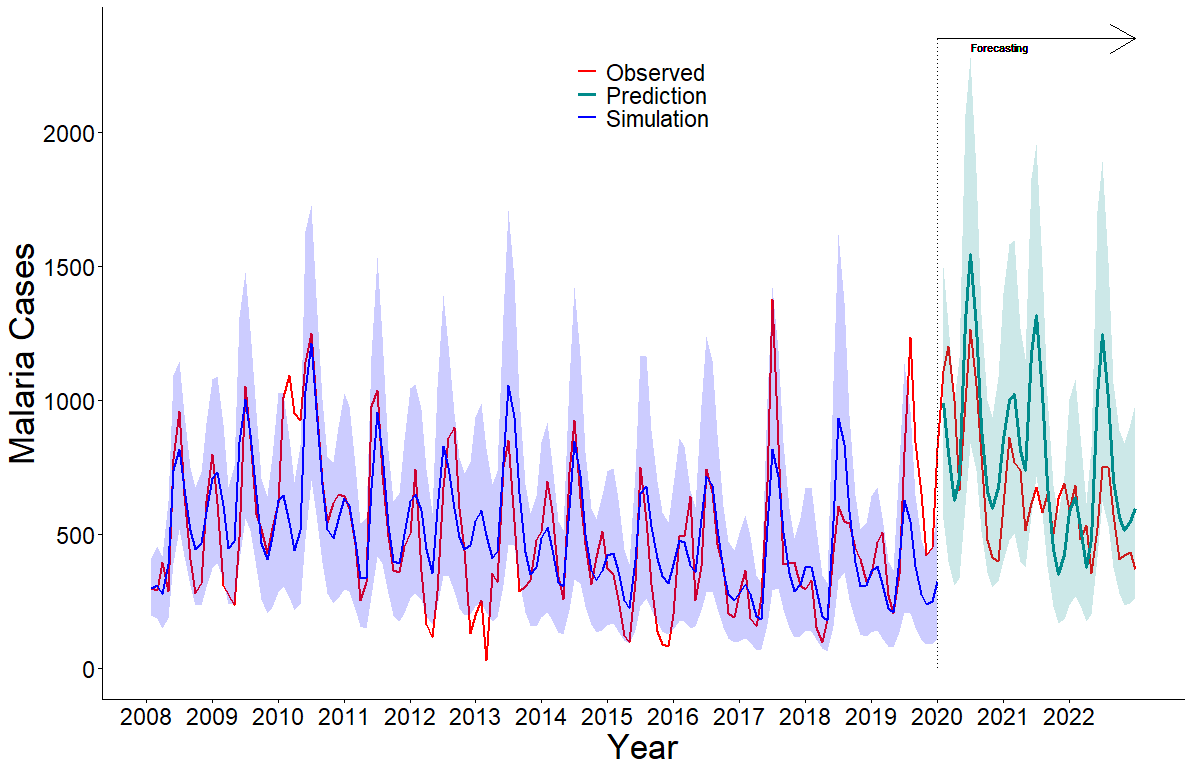

Supplement: S5 Fig — Monthly malaria cases aggregated across the whole study area are depicted in red, and the median of 1000 simulations from the best model (with parameters that maximize the likelihood) is illustrated in blue, with prediction uncertainty (10–90% quantiles) shaded in blue. The median of the forecasted cases for 2020–2022 is shown in cyan, with forecast uncertainty shaded in cyan. (TIFF) [file pcbi.1014004.s005.tiff]

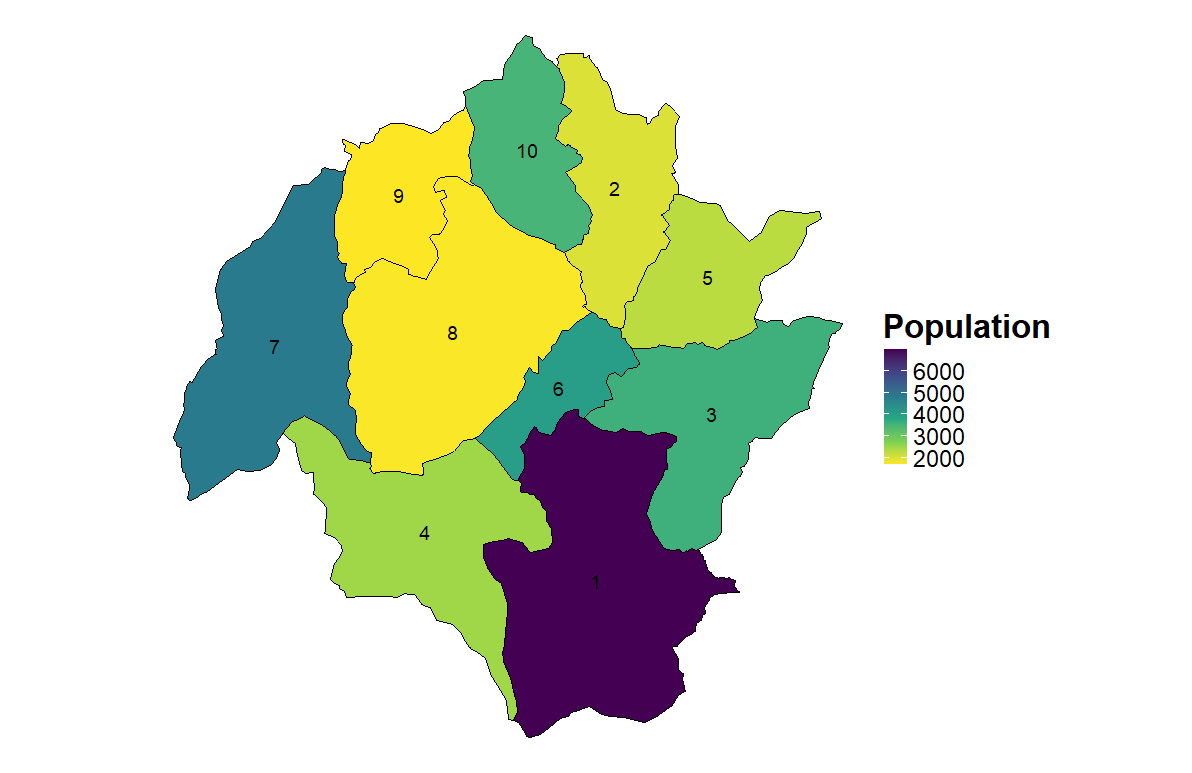

Supplement: S6 Fig — Map of the mean population per cluster during the study period (2008–2020) in the PBIDS surveillance area of western Kenya. The base map is derived from population-based infectious disease surveillance (PBIDS) shapefiles provided by the Kenya Medical Research Institute (KEMRI). The map was generated in R (version 4.4.2) using the ggplot2 package. (TIFF) [file pcbi.1014004.s006.tiff]

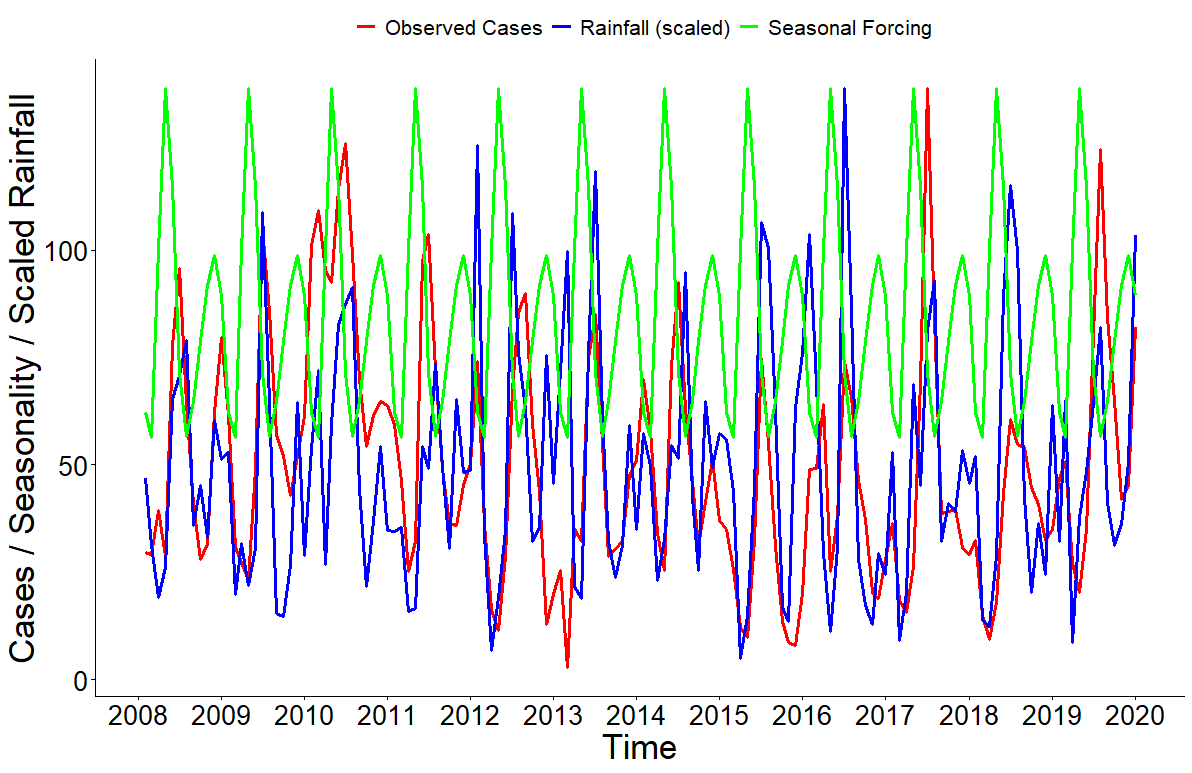

Supplement: S7 Fig — Monthly malaria case counts (red), rainfall with a two-month lag (scaled, blue), and the seasonal component inferred from the model (scaled, green) from 2008–2020. (TIFF) [file pcbi.1014004.s007.tiff]

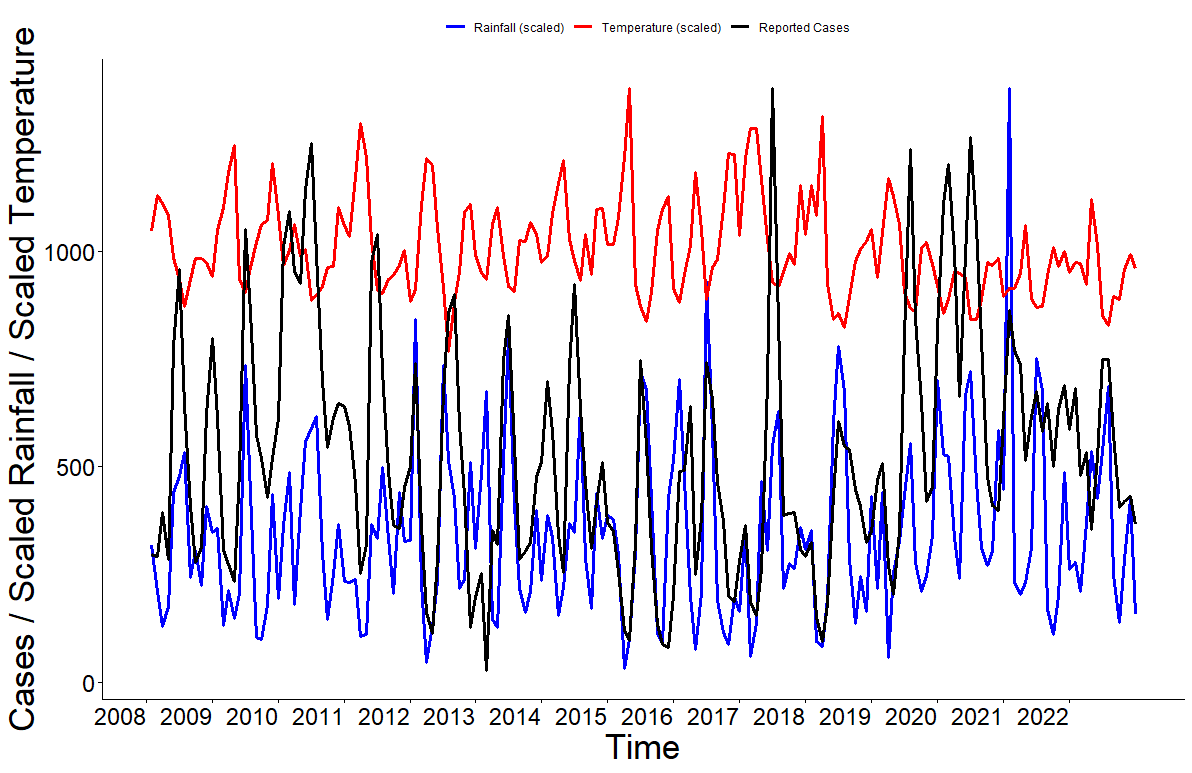

Supplement: S8 Fig — Time series of monthly observed malaria cases (black) and scaled rainfall (blue) for each of the 10 clusters, 2008–2020. (TIFF) [file pcbi.1014004.s008.tiff]

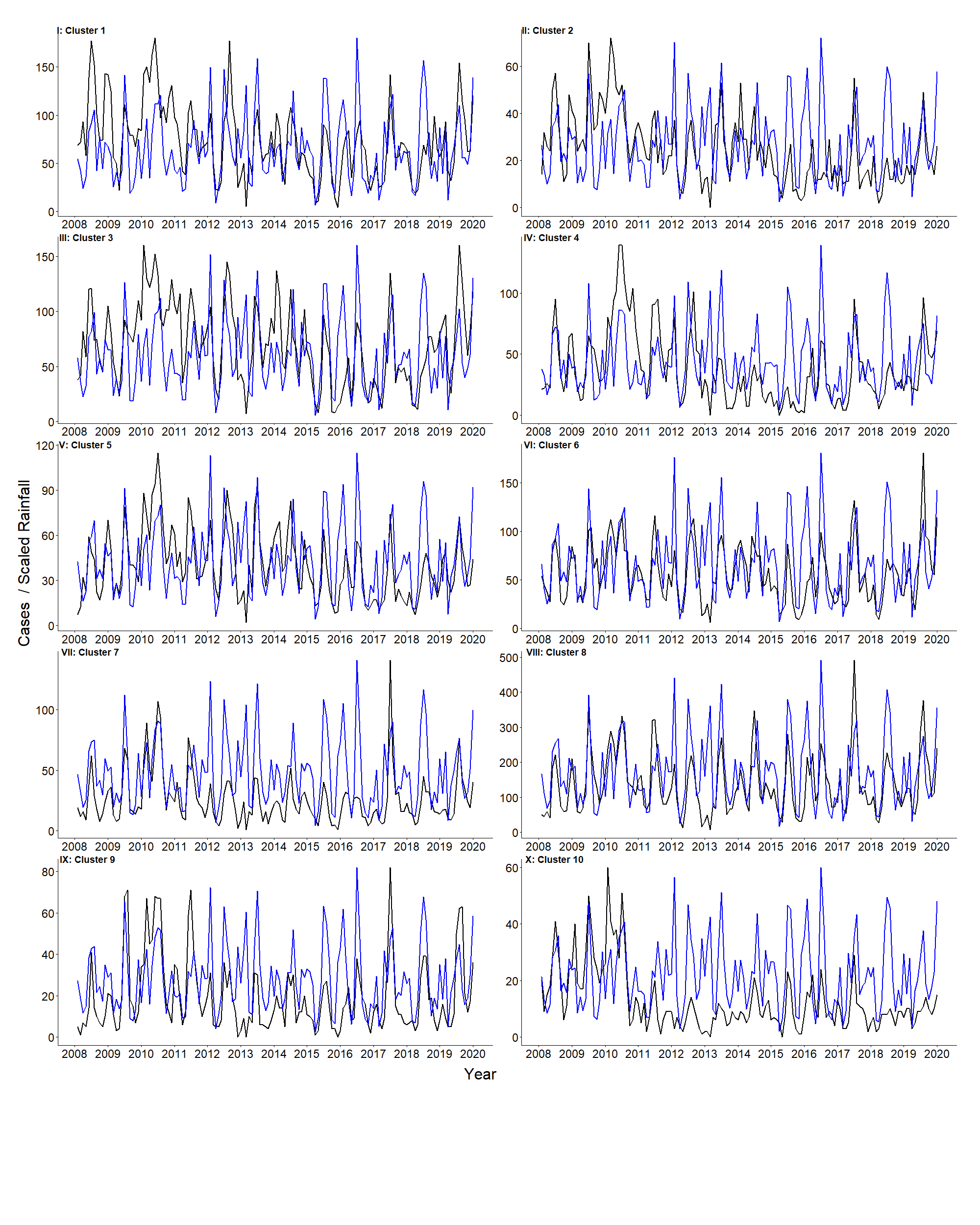

Supplement: S9 Fig — Time series of monthly observed malaria cases (black), scaled rainfall (blue) and scaled temperature (red), 2008–2022. (TIFF) [file pcbi.1014004.s009.tiff]

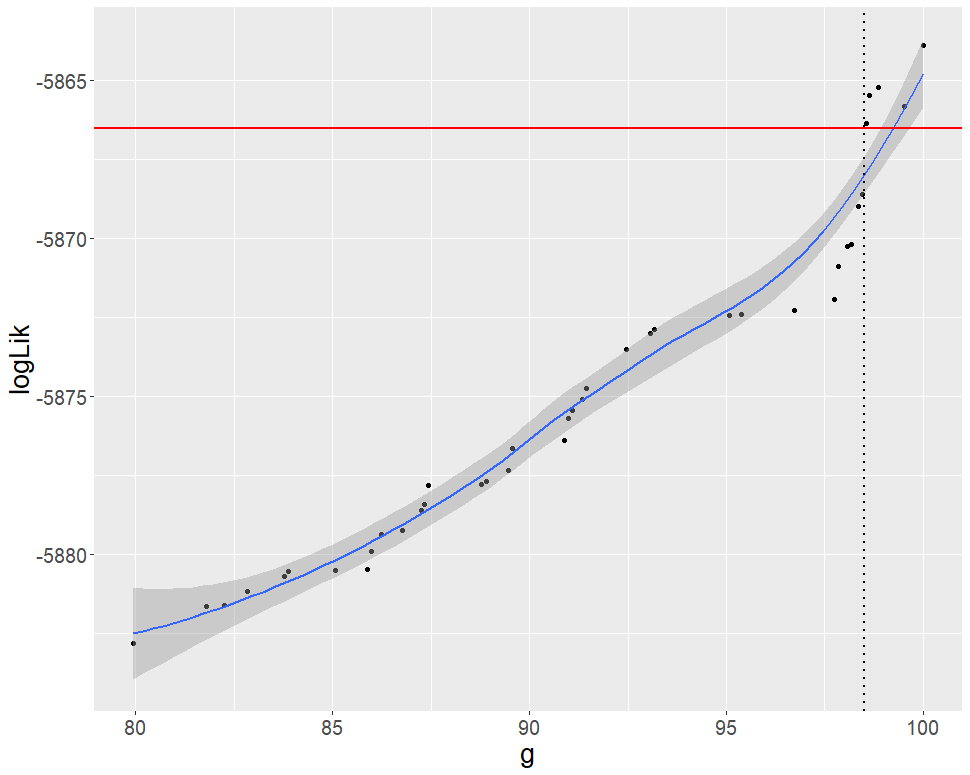

Supplement: S10 Fig — Profile likelihood curve of the parameter g, which governs the strength of spatial coupling, and the corresponding interval (computed as the points at which the profile curve crosses the horizontal line five log-likelihood units below the maximum likelihood estimate). (TIFF) [file pcbi.1014004.s010.tiff]
