# Supplementary material for: Assessing the impact of climate and control interventions on spatio-temporal malaria dynamics using a stochastic metapopulation model
Source: PLoS Comput Biol. 2026 Mar 17;22(3):e1014004. doi: 10.1371/journal.pcbi.1014004 (PMC12995307; doi:10.1371/journal.pcbi.1014004)
Supplement: S1 Table — (PDF) [file pcbi.1014004.s011.pdf]

S1 Table Cluster identification numbers (Cluster ID) and village identification numbers (Village ID) used to identify the villages contained within each cluster.

Cluster ID	Village ID	Village name
1	2	Ongielo
	11	Memba
	12	Mabinju/Asembo
	13	Ndwar/Nyangoma
2	59	Ombulu Masanga
	60	Abuyu
	67	Rambugu B
	68	Got Bondo
3	9	Katombo
	10	Kaminogedo
	19	Powo
	27	Okenye Okiro
4	14	Ujwanga/Nyachida
	15	Mahaya
	35	Sinogo
	36	Tiga
5	24	Saradidi/Miyare
	25	Majango
6	26	Atemo Okenye
	28	Lwak
7	46	Waringa
	50	Riwa Ojelo
	51	Nyabande
	52	Wangarot A
	53	Wangarot B
8	47	Lusi
	48	Ujwanga/Asembo
	49	Sangla
	55	Siger
9	54	Kametho
	58	Gangu
10	56	Rambira
	57	Rambugu A
