# Supplementary material for: Assessing the impact of climate and control interventions on spatio-temporal malaria dynamics using a stochastic metapopulation model
Source: PLoS Comput Biol. 2026 Mar 17;22(3):e1014004. doi: 10.1371/journal.pcbi.1014004 (PMC12995307; doi:10.1371/journal.pcbi.1014004)
Supplement: S2 Table — Starting values for all parameters were [0%, 100%]. (PDF) [file pcbi.1014004.s012.pdf]

S2 Table Fitted reporting rate parameters ρ per cluster in the best malaria spatio-temporal stochastic transmission model. Starting values for all parameters were [0%, 100 %].

**Parameter**	**Cluster ID**	**Estimate**
rho1	1	0.14
rho2	2	0.195
rho3	3	0.24
rho4	4	0.23
rho5	5	0.265
rho6	6	0.385
rho7	7	0.11
rho8	8	0.45
rho9	9	0.28
rho10	10	0.25
total		0.255
