# Supplementary material for: Assessing the impact of climate and control interventions on spatio-temporal malaria dynamics using a stochastic metapopulation model
Source: PLoS Comput Biol. 2026 Mar 17;22(3):e1014004. doi: 10.1371/journal.pcbi.1014004 (PMC12995307; doi:10.1371/journal.pcbi.1014004)
Supplement: S3 Table — Starting values for all parameters were [0%, 100%]. (PDF) [file pcbi.1014004.s013.pdf]

S3 Table Fitted parameters of the susceptible compartment (S) per cluster in the best malaria spatio-temporal stochastic transmission model. Starting values for all parameters were [0%, 100 %].

**Parameter**	**Cluster ID**	**Estimate**
S1	1	12%
S2	2	12.3%
S3	3	9.9%
S4	4	4.3%
S5	5	10%
S6	6	13.1%
S7	7	17.8%
S8	8	12.7%
S9	9	10.7%
S10	10	9.4%
