# Supplementary material for: Assessing the impact of climate and control interventions on spatio-temporal malaria dynamics using a stochastic metapopulation model
Source: PLoS Comput Biol. 2026 Mar 17;22(3):e1014004. doi: 10.1371/journal.pcbi.1014004 (PMC12995307; doi:10.1371/journal.pcbi.1014004)
Supplement: S4 Table — Starting values for all parameters were [0%, 100%]. (PDF) [file pcbi.1014004.s014.pdf]

S4 Table Fitted parameters of the exposed compartment (E) per cluster in the best malaria spatio-temporal stochastic transmission model. Starting values for all parameters were [0%, 100 %].

**Parameter**	**Cluster ID**	**Estimate**
E1	1	9.1%
E2	2	4.8%
E3	3	4.7%
E4	4	4.6%
E5	5	6.6%
E6	6	6.7%
E7	7	6.9%
E8	8	5.5%
E9	9	6.1%
E10	10	5.3%
