# Supplementary material for: Assessing the impact of climate and control interventions on spatio-temporal malaria dynamics using a stochastic metapopulation model
Source: PLoS Comput Biol. 2026 Mar 17;22(3):e1014004. doi: 10.1371/journal.pcbi.1014004 (PMC12995307; doi:10.1371/journal.pcbi.1014004)
Supplement: S5 Table — Starting values for all parameters were [0%, 100%]. (PDF) [file pcbi.1014004.s015.pdf]

S5 Table Fitted parameters of the infectious compartment (I) per cluster in the best malaria spatio-temporal stochastic transmission model. Starting values for all parameters were [0%, 100 %].

**Parameter**	**Cluster ID**	**Estimate**
I1	1	24.4%
I2	2	28.5%
I3	3	26.9%
I4	4	29.6%
I5	5	28.2%
I6	6	22.8%
I7	7	18.1%
I8	8	25.6%
I9	9	25.9%
I10	10	25.7%
