# Supplementary material for: Assessing the impact of climate and control interventions on spatio-temporal malaria dynamics using a stochastic metapopulation model
Source: PLoS Comput Biol. 2026 Mar 17;22(3):e1014004. doi: 10.1371/journal.pcbi.1014004 (PMC12995307; doi:10.1371/journal.pcbi.1014004)
Supplement: S6 Table — Starting values for all parameters were [0%, 100%]. (PDF) [file pcbi.1014004.s016.pdf]

S6 Table Fitted parameters of the asymptomatic compartment with reduced parasitemia (A) per cluster in the best malaria spatio-temporal stochastic transmission model. Starting values for all parameters were [0%, 100 %].

**Parameter**	**Cluster ID**	**Estimate**
A1	1	12.5%
A2	2	10.2%
A3	3	13.4%
A4	4	9.3%
A5	5	12.2%
A6	6	10.4%
A7	7	9.7%
A8	8	7.9%
A9	9	10.6%
A10	10	10.7%
