# Supplementary material for: Assessing the impact of climate and control interventions on spatio-temporal malaria dynamics using a stochastic metapopulation model
Source: PLoS Comput Biol. 2026 Mar 17;22(3):e1014004. doi: 10.1371/journal.pcbi.1014004 (PMC12995307; doi:10.1371/journal.pcbi.1014004)
Supplement: S7 Table — Starting values for all parameters were [0%, 100%]. (PDF) [file pcbi.1014004.s017.pdf]

S7 Table Fitted parameters of the recovered and protected from severe infection compartment (R) per cluster in the best malaria spatio-temporal stochastic transmission model. Starting values for all parameters were [0% , 100 %].

**Parameter**	**Cluster ID**	**Estimate**
R1	1	42%
R2	2	44.2%
R3	3	46.1%
R4	4	52.2%
R5	5	43%
R6	6	47%
R7	7	47.5%
R8	8	48.3%
R9	9	46.7%
R10	10	48.9%
