# Supplementary material for: Assessing the impact of climate and control interventions on spatio-temporal malaria dynamics using a stochastic metapopulation model
Source: PLoS Comput Biol. 2026 Mar 17;22(3):e1014004. doi: 10.1371/journal.pcbi.1014004 (PMC12995307; doi:10.1371/journal.pcbi.1014004)
Supplement: S8 Table — (PDF) [file pcbi.1014004.s018.pdf]

S8 Table Fitted parameters shared across clusters in the best malaria spatio-temporal stochastic transmission model.

**Parameter**	**Description**	**Starting Values**	**Estimate**
spline coefficient ba	B-spline regression coefficient	[-10, 10]	4.18
spline coefficient bb	B-spline regression coefficient	[-10, 10]	0.35
spline coefficient bc	B-spline regression coefficient	[-10, 10]	7.49
spline coefficient bd	B-spline regression coefficient	[-10, 10]	1.66
spline coefficient be	B-spline regression coefficient	[-10, 10]	2.43
spline coefficient bf	B-spline regression coefficient	[-10, 10]	3.67
$1/\mu_{E_u I_u}$	Time from exposed to infected	[1, 365]	27.7 days
$1/\mu_{I_u S_u}$	Time from infected to susceptible	[1, 365]	22.1 days
$1/\mu_{I_u A_u}$	Time from symptomatic to asymptomatic	[1, 365]	197.3 days
$1/\mu_{A_u R_u}$	Time from asymptomatic to recovered	[1, 365]	453.2 days
$1/\mu_{R_u S_u}$	Time from recovered to susceptible	[1, 365]	216.4 days
ψ	Dispersion parameter of the observation noise	[0, 0.5]	0.19
σ	Standard deviation of the process noise	[0, 0.5]	0.27
q	Relative infectivity of partially immune individuals	[0, 1]	0.84
c	Coefficient of reinfection with clinical immunity	[0, 1]	0.15
