# Supplementary material for: Assessing the impact of climate and control interventions on spatio-temporal malaria dynamics using a stochastic metapopulation model
Source: PLoS Comput Biol. 2026 Mar 17;22(3):e1014004. doi: 10.1371/journal.pcbi.1014004 (PMC12995307; doi:10.1371/journal.pcbi.1014004)
Supplement: S9 Table — (PDF) [file pcbi.1014004.s019.pdf]

**S9 Table** Mean absolute error (MAE) and root mean square error (RMSE) between observed malaria cases and the median of 1000 simulated forecasts from the best model.

| Cluster | Mean Absolute Error (MAE) | Root Mean Square Error (RMSE) |
|---|---|---|
| 1 | 142.3 | 200.9 |
| 2 | 140.2 | 195.8 |
| 3 | 148.7 | 209.6 |
| 4 | 144.8 | 204.1 |
| 5 | 148.7 | 208.8 |
| 6 | 142.9 | 202.5 |
| 7 | 146.9 | 207.2 |
| 8 | 141.4 | 199.1 |
| 9 | 145.2 | 208.7 |
| 10 | 147.1 | 206.6 |
