# Supplementary material for: Assessing the impact of climate and control interventions on spatio-temporal malaria dynamics using a stochastic metapopulation model
Source: PLoS Comput Biol. 2026 Mar 17;22(3):e1014004. doi: 10.1371/journal.pcbi.1014004 (PMC12995307; doi:10.1371/journal.pcbi.1014004)
Supplement: S10 Table — Metrics reported include the Akaike information criterion (AIC), mean absolute error (MAE), and root mean square error (RMSE). For the temporal model fitted separately to each region, metrics are summed across regions under the no-coupling assumption (log-likelihood, AIC, MAE, RMSE). (PDF) [file pcbi.1014004.s020.pdf]

S10 Table In-sample performance of models with different covariate sets in the force of infection. Metrics reported include the Akaike information criterion (AIC), mean absolute error (MAE), and root mean square error (RMSE). For the temporal model fitted separately to each region, metrics are summed across regions under the no-coupling assumption (log-likelihood, AIC, MAE, RMSE).

**Model**	**Log-likelihood**	**Nr. of parameters per unit**	**AIC**	**MAE**	**RMSE**
Spatio-temporal model, without covariates	-6211.42	17	12474.84	249.3403	335.4518
Spatio-temporal model, only with seasonality (i.e. splines)	-6090.65	23	12273.5	200.23	280.45
Spation-temporal model with seasonality, LSTD, RAIN and INT covariates	-5577.204	26	12250.47	140.2188	195.8082
Temporal model for every region with seasonality, LSTD, RAIN and INT covariates	-5915.29	26	12348.97	161.25	219.88
